# Immunometabolism of Phagocytes During *Mycobacterium tuberculosis* Infection

**DOI:** 10.3389/fmolb.2019.00105

**Published:** 2019-10-14

**Authors:** Ranjeet Kumar, Pooja Singh, Afsal Kolloli, Lanbo Shi, Yuri Bushkin, Sanjay Tyagi, Selvakumar Subbian

**Affiliations:** Public Health Research Institute, New Jersey Medical School, Rutgers, The State University of New Jersey, Newark, NJ, United States

**Keywords:** tuberculosis, Mycobacteria, innate immunity, immunometabolism, immune cells, epigenetics, metabolic regulators, infection

## Abstract

Tuberculosis (TB), caused by *Mycobacterium tuberculosis* (Mtb) remains as a leading killer among infectious diseases worldwide. The nature of the host immune response dictates whether the initial Mtb infection is cleared or progresses toward active disease, and is ultimately determined by intricate host-pathogen interactions that are yet to be fully understood. The early immune response to infection is mediated by innate immune cells, including macrophages and neutrophils that can phagocytose Mtb and mount an antimicrobial response. However, Mtb can exploit these innate immune cells for its survival and dissemination. Recently, it has become clear that the immune response and metabolic remodeling are interconnected, which is highlighted by the rapid evolution of the interdisciplinary field of immunometabolism. It has been proposed that the net outcome to Mtb infection—clearance or chronic disease—is likely a result of combined immunologic and metabolic activities of the immune cells. Indeed, host cells activated by Mtb infection have strikingly different metabolic requirements than naïve/non-infected cells. Macrophages activated by Mtb-derived molecules or upon phagocytosis acquire a phenotype similar to M1 with elevated production of pro-inflammatory molecules and rely on glycolysis and pentose phosphate pathway to meet their bioenergetic and metabolic requirements. In these macrophages, oxidative phosphorylation and fatty acid oxidation are dampened. However, the non-infected/naive, M2-type macrophages are anti-inflammatory and derive their energy from oxidative phosphorylation and fatty acid oxidation. Similar metabolic adaptations also occur in other phagocytes, including dendritic cells, neutrophils upon Mtb infection. This metabolic reprogramming of innate immune cells during Mtb infection can differentially regulate their effector functions, such as the production of cytokines and chemokines, and antimicrobial response, all of which can ultimately determine the outcome of Mtb-host interactions within the granulomas. In this review, we describe key immune cells bolstering host innate response and discuss the metabolic reprogramming in these phagocytes during Mtb infection. We focused on the major phagocytes, including macrophages, dendritic cells and neutrophils and the key regulators involved in metabolic reprogramming, such as hypoxia-inducible factor-1, mammalian target of rapamycin, the cellular myelocytomatosis, peroxisome proliferator-activator receptors, sirtuins, arginases, inducible nitric acid synthase and sphingolipids.

## Introduction

Tuberculosis (TB) remains as significant cause of mortality and morbidity among infectious diseases around the world. In 2017, an estimated 10 million people developed active disease, while 1.6 million died from TB (WHO, 2018). About a third of the world's population is believed to be latently infected with *Mycobacterium tuberculosis* (Mtb) without obvious disease symptoms. Of these, 5–10% will develop active TB in their lifetime, if/when their host immunity wanes (WHO, 2018).

The host immune response during the various stages of Mtb infection is complex and not fully understood. Studies on human patients and experimental animal models of TB have shown that phagocytes such as macrophages and neutrophils are the primary immune effector cells against Mtb (Keane et al., 2001; North and Jung, 2004; Alcais et al., 2005). Engagement of surface receptors in these cells with mycobacterial cell wall molecules such as lipoarabinomannan and secreted proteins ultimately activates the immune cells to secrete a plethora of cytokines and chemokines, which aid in the recruitment of other leukocytes from circulation to the site of infection. A hallmark of successful initial Mtb infection is the formation of granuloma in the infected tissues, which is an organized cellular structure comprised of a variety of innate and adaptive immune cells (Ramakrishnan, 2012). Mature macrophages, characterized by an increased cytoplasm-to-nucleus ratio and larger number of organelles, are capable of developing into multinucleated giant cells as well as foam cells that accumulate lipid bodies. These cells are critical constituents of TB granulomas, which undergo structural changes over time (Russell et al., 2009; Guerrini et al., 2018). Initial development of granuloma is marked by its extensive vascularization through vascular endothelial growth factor (VEGF) mediated responses, leading to recruitment of macrophages, lymphocytes, and DCs, into the site of infection (Caceres et al., 2009). Further structural changes in granuloma are marked with the presence of different morphotypes of macrophages, including multinucleated giant cells, epithelioid cells, and foamy macrophages (Murphy, 2001; Ordway et al., 2005; D'Avila et al., 2006). The later cells are generated as a result of intracellular accumulation of lipid droplets consisting of neutral lipids, mainly cholesteryl esters (CE) and/or triglycerides (TAG) surrounded by a monolayer of phospholipids comprising structural proteins, cholesterol and enzymes (Martin and Parton, 2006; Saka and Valdivia, 2012; Guerrini et al., 2018). Accumulation of TAG, a significant constituent of foam cells within the granuloma, in Mtb-infected human primary macrophages requires tumor necrosis factor receptor (TNFR) signaling, activation of caspase cascade and mTORC1 (Russell et al., 2009). Rupture of foam cells due to exacerbated infection and/or inflammation and the release of their contents likely sustains the disease pathology and generation of caseum, which leads to progressive destruction of lung tissues (Russell et al., 2009). Foamy macrophages (FM) are also reported to provide Mtb their physiological niche similar to non-replicative vegetative state (Russell et al., 2009). In response to strong anti-mycobacterial response, the structure tends to become stratified, and a fibrous cuff is formed delineating central macrophages rich region from peripheral lymphocytic milieu (Russell et al., 2009). In majority of cases, granuloma maintains a dynamic state where Mtb is contained, and disease is resolved. However, in some cases there is increased accumulation of caseum in central region of granuloma, which leads to necrosis of cells and subsequent release of Mtb into the airways (Russell et al., 2009).

Other cell types that are present in the granulomas are neutrophils, dendritic cells (DCs), various subtypes of B and T lymphocytes, natural killer cells, fibroblasts, and epithelial cells (Ramakrishnan, 2012). Findings from rabbit and non-human primate models of Mtb infection, which are immunopathologically “relevant-to-human,” have shown that granulomas contain a macrophage-rich core with necrosis of infected cells surrounded by a lymphocytic cuff, consisting mainly of T and B cells (Capuano et al., 2003; Subbian et al., 2011, 2013; Mattila et al., 2013). However, findings in human pulmonary TB cases and relevant experimental animal models have revealed many different types of granulomas based on the presence and arrangement of various immune cell types. These divergent types include highly cellular, “solid” granulomas, caseating, necrotic and non-necrotic, fibrotic nodules, suppurative (comprising a surge of neutrophils), and closed and open cavitary lesions (Capuano et al., 2003; Subbian et al., 2011, 2013; Mattila et al., 2013). These granulomas vary in size, maturation state, and the quantity and quality of the resident immune cells. Partially mineralized and highly fibrotic (fibrocalcific) granulomas are primarily found in latent infection, while the number and cellular complexity of granulomas is of a higher order during active TB (Gideon and Flynn, 2011). However, different types of granulomas have been reported in the tissues of active TB and latent infection, making it difficult to assess the infection status of a host-based on the type of granulomas present in the organ (Mattila et al., 2013).

Although granulomas are thought to restrict bacterial growth and expansion by “walling off,” Mtb can exploit the immune cells within and outside of the granuloma for its persistence, proliferation, and dissemination (Davis and Ramakrishnan, 2009; Volkman et al., 2010). Based on the intricate interaction between the host immune response and bacterial determinants that are yet to be fully understood, the infecting Mtb is either contained and/or cleared or replicates and disseminates in the granuloma. The outcome of this interaction also results in a spectrum of clinical manifestations in the infected host, ranging from symptomatic active disease to sub-clinical, incipient, percolator, and intermittent disease to persistent, asymptomatic latent infection (Vynnycky and Fine, 2000).

The infection of host immune cells by Mtb causes several changes including metabolic reprogramming, which differentially regulates various cytokines and chemokines associated with clearance, containment, or progression of Mtb infection in host cells (Olakanmi et al., 2002; Gleeson et al., 2016; Qualls and Murray, 2016). In particular, a shift in glucose and lipid metabolism is critical for defining the fate of host cell function in the context of mycobacterial survival within the granuloma (Shi et al., 2016). Rapid advances in the field of immunology and metabolism have given rise to an interdisciplinary field termed immunometabolism (Mathis and Shoelson, 2011). Core metabolic processes such as glycolysis, Krebs cycle, fatty acid metabolism, and nitrogen metabolism are vital for the proper functioning of any cell (DeBerardinis and Thompson, 2012; Escoll and Buchrieser, 2018). In general, cells in a “resting” condition mostly rely on oxidative phosphorylation (OXPHOS) to generate ATP from NADH, and FADH made during the Krebs cycle. However, under oxygen-depleting or hypoxic conditions and during high-energy requirements, the cells switch to aerobic glycolysis to generate ATP (Escoll and Buchrieser, 2018). Although aerobic glycolysis produces ATP less efficiently than OXPHOS, it produces more ATP at a faster rate, which meets the high energy demands of the immune cells. Apart from energy needs, aerobic glycolysis also provides the precursors for chemical constituents, such as nucleotides, amino acids, and lipids (Lunt and Vander Heiden, 2011). Therefore, metabolic reprogramming fulfills both the bioenergetic and biosynthetic needs of cells for rapid proliferation, differentiation, and/or production of secretory components (DeBerardinis and Thompson, 2012; Escoll and Buchrieser, 2018).

Metabolic reprogramming in immune cells is controlled by host-derived transcriptional regulators such as hypoxia-inducible factor (HIF), mechanistic/mammalian target of rapamycin (mTOR), myelocytomatosis virus oncogene (Myc), and glycogen synthase kinase 3 (GSK3) (Wahl et al., 2012; O'Neill and Pearce, 2016; Jellusova and Rickert, 2017) (Table 1). Besides, immune cells can initiate metabolic reprogramming through signaling pathways elicited in response to host-pathogen interactions through receptors, such as pattern recognition receptors (PRR), cytokine receptors, and antigen receptors (Escoll and Buchrieser, 2018). In these cases, the onset of aerobic glycolysis is essential for immune cell activation and corresponding production of cytokines, chemokines, and antibacterial molecules. Therefore, the metabolic needs of an infected and/or activated immune cell are significantly different from a resting or non-infected cell. Taken together, recent advances focused on the immunometabolism during microbial infections have provided new insights into host immune regulation mechanisms, opening up new avenues for the development of host-directed therapies to enhance treatment outcomes (Eisenreich et al., 2019; Prusinkiewicz and Mymryk, 2019; Wang et al., 2019).

**Table 1:** Key metabolic regulators that modulate immune responses.

**Metabolic regulators**	**Mechanism of action**	**Biological effect**	**References**
HIF-1α	Upregulates expression of glycolytic enzymes and IL-1β	Increases glycolysis and Th1 immune response	Nizet and Johnson, 2009; Shi et al., 2015; Braverman et al., 2016
mTORC1	Activates SREBP/MYC signaling pathway	Augments NK cell development and its activation	Donnelly et al., 2014; O'Brien et al., 2019
cMyc	Induces cell proliferation, and glucose and glutamine metabolism	Regulates inflammatory response	Stine et al., 2015
GSH	Induces mTOR and NFAT activities, and promotes glycolysis and glutaminolysis in activated T cells	Augments T cell growth, activates T cell-mediated inflammatory response and fine-tunes innate immunity during infections	Diotallevi et al., 2017; Mak et al., 2017
Sirtuins	Inhibit NF-κB signaling and glucose metabolism	Suppress inflammation and regulate immuno-pathogenesis during infection	Vachharajani et al., 2016; Cheng et al., 2017
PPAR-γ	Upregulates PGE2 production and inhibits NF-κB signaling	Enhances lipid droplet formation and suppresses the pro-inflammatory response	Almeida et al., 2012; Salamon et al., 2014
PPAR-α	Activates TFEB signaling and promotes fatty acid oxidation	Downregulates lipid accumulation and augments the innate immune response	Kim et al., 2017
Arginase-1	Downregulates production of NO and RNS	Augments macrophage polarization toward M2 and promotes pathogen survival	Duque-Correa et al., 2014
Arginase-2	Activates the LXR-mediated anti-inflammatory signaling pathway	Suppresses macrophage immunity during infection	Lewis et al., 2011; Koo et al., 2012
iNOS	Promotes NO production and nitrosylates enzymes involved in metabolic pathways	NO exerts microbicidal activities, inhibits mitochondrial respiration, and promotes a metabolic shift to glycolysis and fatty acid oxidation	Braverman and Stanley, 2017
Sphingolipids (S1P, C1P) and associated enzymes (SK1)	Promotes cell membrane integrity and phagosome maturation	Stimulates macrophage activation, cell repair, and division, and a shift to lipid metabolism in granulomas	Malik et al., 2003; Liu et al., 2010

In this review, we describe the role of metabolic reprogramming in various phagocytic cells that bolster host innate immune response and their relevance to Mtb infection. Since a full report on the host immune response and the cells involved is beyond the scope of this article, we focus on major phagocytes and the key regulators that control metabolic reprogramming in these cells during Mtb infection.

## Primary Phagocytes Involved in TB Pathogenesis

### Macrophages

**Figure 1:**
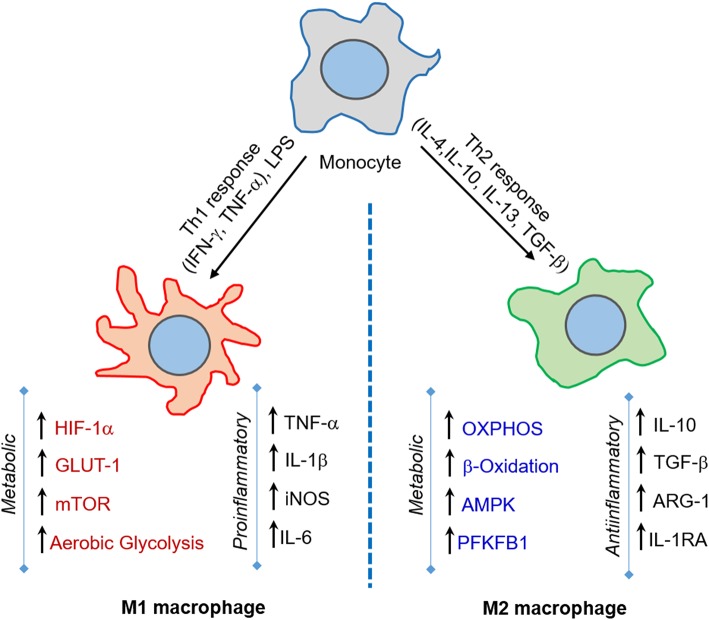
M1 and M2 macrophages orchestrate host immunity in response to a variety of stimuli. Macrophages are activated to M1-type upon challenge with pro-inflammatory IFN-γ/LPS or microbial infection, while M2-type macrophages are induced upon IL-4/IL-10/IL-13 or TGF-β exposure. The M1 state is marked by increase in aerobic glycolysis induced by HIF-1α, GLUT-1, and mTOR expression, and is involved in microbicidal activities. In contrast, the M2 state is marked by OXPHOS and enhanced lipid metabolic activities, such as fatty acid oxidation, and predominantly elicits an anti-inflammatory response and tissue repair.

Macrophages shape the nature and course of host immunity against Mtb infection by playing a crucial early role, such as phagocytosis of Mtb and triggering an antimicrobial response (van Crevel et al., 2002; Gutierrez et al., 2004). Although macrophages elicit an early host protective immunity against Mtb, these innate cells are also thought to provide an intracellular niche where Mtb thrives (Schluger and Rom, 1998). Macrophages demonstrate a high level of heterogeneity in morphology and function (Austyn and Gordon, 1981; Kaplan and Gaudernack, 1982). Based on the heterogeneity observed mainly in *in vitro* cell culture systems, macrophages are categorized into four major groups: type I, type II, alternatively activated and deactivated macrophages (Figure 1) (Gordon, 2003; Gordon and Taylor, 2005). Type I macrophages are activated through lymphoid cell mediators such as IFN-γ and LPS, while type II is differentiated upon ligation of receptors by immune complexes (Guirado et al., 2013). Both of these macrophage types possess high microbicidal activity by producing pro-inflammatory cytokines such as TNF-α, IL-1β, and IL-6, and reactive oxygen species (ROS) and inducible nitric oxide synthase (iNOS), which leads to the production of nitric oxide (NO) (Bogdan, 2001). The third group, alternatively activated macrophages, is generated in response to Th-2 type cytokines such as IL-4 or IL-13 (Gordon, 2003). These macrophages secrete anti-inflammatory cytokines such as IL-10 and TGF-β and are mostly involved in tissue repair and humoral immunity (Gordon, 2003; Mosser, 2003). Finally, deactivated macrophages are produced in response to anti-inflammatory cytokines IL-10 or TGF-β, and are involved in the production of anti-inflammatory cytokines and prostaglandin E2, and have reduced MHC II expression (Guirado et al., 2013).

One of the initial events following inhalation of Mtb is phagocytosis by alveolar macrophages that secrete IL-8 and facilitate the recruitment of neutrophils to the infection site (Schlesinger, 1996). Various host cell receptors, including complement receptors, C-type lectins, mannose receptors, surfactant proteins (SpA), and CD14, are involved in the uptake of Mtb by macrophages (Kleinnijenhuis et al., 2011). The association between the type of receptor involved in phagocytosis and the fate of engulfed Mtb within the macrophages remains poorly understood. The nature of the engulfing receptor can influence the downstream signaling and subsequent processing of the bacilli (Sanchez et al., 2010; Marakalala and Ndlovu, 2017; Queval et al., 2017). Thus, Mtb phagocytosed by specific cell surface receptors is thought to be processed differentially that can favor intracellular bacillary growth, while engagement and signaling by other receptors destroy the bacteria (Killick et al., 2013; Mortaz et al., 2015; Liu et al., 2017). Ultimately, the fusion of mycobacteria-containing phagosomes with lysosomes results in bacterial killing within the phagocyte. Similarly, host cell production of ROS and nitrogen (RNS) intermediates facilitate intracellular bacterial killing (MacMicking et al., 1997; Cooper et al., 2000; Ehrt and Schnappinger, 2009). Besides, macrophages phagocytose and eliminate Mtb through autophagy and apoptosis (Eruslanov et al., 2005; Wolf et al., 2007; Eum et al., 2010; Behar et al., 2011; Lowe et al., 2012). In general, these antibacterial mechanisms of phagocytes are successful in eliminating non-virulent bacillus; however, virulent Mtb strains have developed mechanisms to bypass these efforts, by preventing phagolysosome fusion, and surviving in the presence of ROS and RNS, thereby adapting to the hostile intracellular environment (Sturgill-Koszycki et al., 1994). Successful pathogenesis by Mtb further relies on subverting host cell death pathways; for example, Mtb inhibits host cell apoptosis and induces necrosis as a strategy to survive intracellularly and disseminate to other cells (Hinchey et al., 2007; Behar et al., 2011; Welin et al., 2011; Wong and Jacobs, 2011).

German Nobel laureate Otto Heinrich Warburg first described aerobic glycolysis for tumor cells in the 1920s (Warburg, 1956). Recent reports have revealed that Mtb can induce a Warburg-like metabolic reprogramming in infected cells both *in vivo* and *in vitro* (Shin et al., 2011; Somashekar et al., 2011; Appelberg et al., 2015; Shi et al., 2015; Gleeson et al., 2016; Lachmandas et al., 2016a,b; Billig et al., 2017). This effect is characterized by classical upregulation of glucose uptake and glycolysis-coupled deviation of glycolytic intermediates to the synthesis of large lipid bodies, which accumulate in the macrophages (Kelly and O'Neill, 2015). Fatty acids in these lipid bodies of infected macrophages provide a nutritional niche for the intracellular bacteria (Lee et al., 2013; Podinovskaia et al., 2013; VanderVen et al., 2015; Nazarova et al., 2017). It has also been reported that induction of aerobic glycolysis by Mtb in human monocyte-derived macrophages and primary human alveolar macrophages are required for the optimal production of IL-1β and suppression of the anti-inflammatory cytokine IL-10, leading to reduced bacterial load (Gleeson et al., 2016).

One of the critical virulent factors of pathogenic Mtb, ESAT-6, stimulates translocation of the glucose transporters Glut-1 and Glut-3 from the cytosol to the cell membrane, which correlates with enhanced uptake of glucose into human macrophages (Singh et al., 2015). Glucose uptake by THP-1 macrophages increases proportionately with the virulence of the Mtb strain, supporting a key metabolic function in Mtb progression (Singh et al., 2015). In addition, augmentation of aerobic glycolysis promoted activation of the cellular apoptotic response upon Mtb infection (Matta and Kumar, 2016). However, Mtb adapts to the host cell environment by switching its central carbon metabolism (CCM) toward catabolism of host lipid substrates, which includes the pathogen-induced lipid bodies accumulated within the macrophages (Lee et al., 2013). Recent studies also suggest that this lipid body accumulation in human macrophages relies on the induction of *de novo* cholesterol and fatty acid synthesis (FAS), and the generation of ketone body D-3-hydroxybutyrate by the host cell (Kim et al., 2010; Singh et al., 2012). Although Mtb ESAT-6 is involved in the initiation of lipid body formation, BCG, a non-pathogenic vaccine strain of *M. bovis* can also induce lipid bodies in infected macrophages (Melo and Dvorak, 2012). Therefore, lipid body formation may be a general consequence of mycobacterial infection rather than playing a key role in TB pathogenesis.

An important metabolic regulator, PPAR-γ, is highly upregulated in human and mouse macrophages upon mycobacterial infection (Almeida et al., 2009; Lagranderie et al., 2010; Rajaram et al., 2010). The increased expression of PPAR-γ leads to enhanced lipid droplet formation inside macrophages and down-modulation of immune responses against Mtb (Rajaram et al., 2010; Mahajan et al., 2012). This observation suggests that increased PPAR-γ expression is a strategy adopted by Mtb to establish infection in the host. Consistent with this, inhibition of PPAR-γ expression in macrophages resulted in enhanced mycobacterial killing (Almeida et al., 2012).

Different lineages of macrophages have differential responses to Mtb infection (Epelman et al., 2014; Gibbings et al., 2015; van de Laar et al., 2016), although macrophages derived from different ontogenies coexist in several tissues. A recent study in mice showed that alveolar macrophages are committed to fatty acid oxidation, which serves as a favorable niche for Mtb, while interstitial macrophages are glycolytically active, restricting bacterial growth (Huang et al., 2018). Further, studies using Mtb-infected mouse bone marrow-derived macrophages (BMDM) treated with 2-DG (2-D-Glucose, a glycolysis inhibitor) or etomoxir (ETO; a fatty acid oxidation inhibitor) showed that inhibition of glycolysis enhanced bacterial growth, while inhibition of fatty acid oxidation reduced bacterial count (Huang et al., 2018). Thus, it was proposed that restricting alveolar macrophages and increasing the number of interstitial macrophages might be an effective strategy to combat Mtb.

Activation of macrophages by signals emerging from Mtb infection leads to their polarization toward an M1 state. These cells undergo a metabolic shift toward aerobic glycolysis and arginine metabolism and express inflammatory cytokines such as TNF-α, IL-1β, IFN-γ, and IL-12 (McClean and Tobin, 2016) (Figure 1). During the advanced disease stage, the macrophages adapt to an M2- polarization state, whereby metabolic reprogramming leads to a dependence on oxidative phosphorylation to supply energy needs and the expression of anti-inflammatory cytokines such as IL-4, IL-10, and TGF-β (McClean and Tobin, 2016). The M2-polarized state is also partly required to maintain homeostasis and prevent self-destruction from an over-active immune response (O'Neill and Pearce, 2016; Escoll and Buchrieser, 2018). However, a study using computational genome-scale metabolic models predicted that Mtb decelerates OXPHOS and glycolysis in human alveolar macrophages, which results in decreased ATP production (Bordbar et al., 2010). This study also predicted that BCG and other non-pathogenic or dead mycobacteria could accelerate glycolysis and OXPHOS (Bordbar et al., 2010). In this study, a reduced glycolytic proton efflux rate was observed in Mtb-infected hMDMs and THP-1 macrophages. In contrast, an increased glycolytic proton efflux rate was noted in macrophages upon BCG infection. Therefore, reducing the glycolytic rate could be a strategy through which Mtb subverts the host immune response. It was also proposed that citrate is involved in this glycolysis reduction. Because citrate is an inhibitor of phosphofructokinase, an enzyme responsible for the conversion of fructose-6-phosphate to fructose-1, 6 biphosphate, and ADP, which is a rate-limiting step in glycolysis, predictions from this study challenge the model that Mtb infection induces the Warburg effect in macrophages (Bordbar et al., 2010).

In addition to glucose and fatty acid metabolism, amino acid metabolism has also been investigated in the context of Mtb pathogenesis. In a recent study, it was observed that in murine and macaque macrophages, as well as in their lungs, Mtb induced the expression of indoleamine 2, 3 dioxygenases (IDO), an enzyme involved in tryptophan catabolism (Mehra et al., 2013; Gautam et al., 2018). In these experimental models, suppression of IDO activity resulted in reduced bacterial load and increased host survival (Gautam et al., 2018). IDO catabolizes tryptophan (Trp) to Kynurenine (Kyn) and other metabolites. It also suppresses the host immune response, particularly IFN-γ production by CD4+ T cells (Mbongue et al., 2015). Since Mtb can synthesize its own Trp *de novo* in infected host phagocytes, the production of IDO by the host cells have less effect on intracellular Mtb metabolism; yet both Mtb-Trp and host-IDO impact the protective host immune response. Indeed, IDO is particularly enriched in the macrophage-rich, inner layer of granulomas in the lungs of Mtb-infected non-human primates (Mehra et al., 2013). Inhibition of IDO activity [e.g., using 1-methyltryptophan (1-MT, D-1MT)] was also associated with reorganization of the granuloma that allowed lymphocytic trafficking into the macrophage-tropic internal layers (Gautam et al., 2018). Taken together, macrophages, as the primary phagocytes of Mtb infection, can rewire their metabolic pathways upon infection and/or activation through multiple mechanisms, which are yet to be fully explored.

### Dendritic Cells

**Figure 2:**
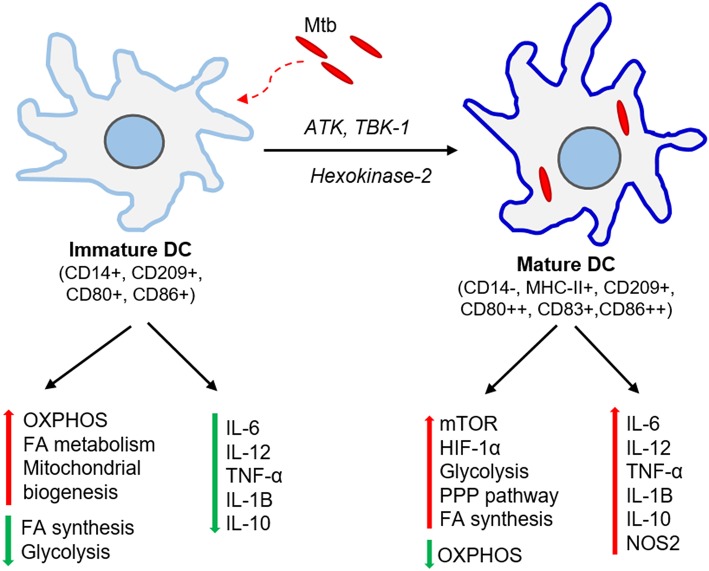
Immunometabolic changes associated with DC maturation. Immature DCs produce no or meager amount pro-inflammatory cytokines. These DCs have elevated oxidative phosphorylation (OXPHOS), fatty acid (FA) metabolism and mitochondrial biogenesis while dampening FA synthesis and glycolysis. Mtb infection activates ATK, TBK-1, and hexokinase-2 signaling pathways and induces DC maturation. The mature DCs express higher levels of major histocompatibility complex II (MHC-II) and co-stimulatory molecules (CD80, CD83, CD86). These DCs up-regulate molecules involved in mTOR and HIF-1α signaling pathways, elevate glycolysis, fatty acid synthesis and pentose phosphate pathway (PPP), and dampens OXPHOS. Mature DCs also produce significant amount pro-inflammatory cytokines such as IL-6, TNF-α, IL-1β.

Dendritic cells (DC) serve as a bridge between the host innate and adaptive immune response during Mtb infection (Mellman and Steinman, 2001; Kapsenberg, 2003). DCs carrying live Mtb and/or its antigens migrate to the regional lymph nodes where they prime T cells, leading to generation and proliferation of effector and memory T cells (Gallegos et al., 2008; Reiley et al., 2008; Wolf et al., 2008). Migration of DCs to lymph nodes is facilitated by IL-12-p40-dependent mechanisms and upregulation of CCR-7 (Khader et al., 2006). DCs are crucial for mounting CD4+ T cell response involving Th1 cytokines, such as IFN-γ and TNF-α (Tascon et al., 2000; Humphreys et al., 2006). The depletion of CD11C+ cells, which includes DCs, delays the CD4+T cell response in mice infected with Mtb (Tian et al., 2005). This shows that the early interaction of DCs with Mtb shapes the development of subsequent adaptive immune response (Figure 2). Since DCs are a heterogeneous cell population, the effectiveness of immune responses against Mtb is coordinated among the different subtypes. Lai et al. recently found in a mouse model that migratory CD11b+ DCs are involved in Th1 priming and activation following Mtb infection, while their counterpart, CD103+ DCs, plays a regulatory role through production of IL-10 and inhibiting priming of Th1 cells (Lai et al., 2018). It has also been reported that treatment of DCs with glutathione (GSH) precursor *in vitro* enhanced their ability to inhibit Mtb by innate immune mechanisms, and facilitated an efficient adaptive immune response (Morris et al., 2013a). However, several microbial factors, including cell wall components and secretion products, can interfere with DC-T cell signaling *in vivo* (Ting et al., 1999; Fortune et al., 2004; Reed et al., 2004; Banaiee et al., 2006; Pathak et al., 2007). Recently, Mtb Hip1 was identified as a critical factor that impairs DC function; infection of DCs with *hip1*-mutant Mtb resulted in significantly higher expression of pro-inflammatory cytokines such as IL-12 and enhanced surface expression of MHCII, CD4, and CD86, which are required for DC maturation (Madan-Lala et al., 2014).

The metabolic requirements for DCs are distinct among different subtypes, tissues, and stages of maturation (Figure 2). Bone marrow-derived dendritic cells (BMDCs) can adopt Warburg effect, i.e., aerobic glycolysis, upon stimulation with TLR agonists, leading to optimal maturation and effector functions in both human and mouse DCs (Krawczyk et al., 2010). This induction of glycolysis is regulated by PI3K/TBK1/IKKε/AKT signaling, which promotes rapid translocation of Hexokinase-2 to mitochondria, facilitating glucose catabolism required for DC maturation (Miyamoto et al., 2008; van der Windt et al., 2012). The by-products of glucose metabolism are used in pentose phosphate pathway (PPP) metabolism, lactate production, and fatty acid synthesis. This metabolic reprogramming regulates the breadth and depth of adaptive immune response induced by the DCs (Pearce and Everts, 2015). One of the essential regulators of DC metabolic remodeling, mTOR, is activated upon interaction of mycobacterial pathogen-associated molecular pattern (PAMP) molecules with DC surface receptors, which promotes protein synthesis and cell growth (Pearce and Everts, 2015).

Alteration in fatty acid metabolism of DCs in response to Mtb infection is another area of intense research. Differentiation of human monocytes to DCs in response to GM-CSF and IL-4 is accompanied by increased expression of the peroxisome proliferator-activated receptor-gamma (PPAR-γ), a transcription factor involved in lipid metabolism that is also upregulated in macrophages in response to Mtb infection (Le Naour et al., 2001; Ishikawa et al., 2007). Monocyte differentiation to DCs is dependent on phosphoinositol-3 kinase (PI3K)-mediated activation of the mTOR complex1 (mTORC1) that in turn activates its downstream target PPAR-γ, which affects cell maturation and function by controlling lipid metabolism (Nencioni et al., 2002; Szatmari et al., 2004, 2007; Gogolak et al., 2007). Further, inhibiting cytosolic fatty acid synthase (FASN) by blocking acetyl CoA-carboxylase (AAC1) reduced monocyte-derived dendritic cell (moDC) differentiation (Rehman et al., 2013). Differentiated moDCs contain higher numbers of mitochondria, show a higher oxygen consumption rate (OCR), and produce more ATPs than other non-differentiated monocytes (Del Prete et al., 2008; Zaccagnino et al., 2012). Consistent with this, moDC differentiation can be partially prevented by blocking the mitochondrial electron transport chain (ETC) with complex-I inhibitor, rotenone (Del Prete et al., 2008; Zaccagnino et al., 2012). Thus, differentiation of DCs from human monocytes is governed by OXPHOS and fatty acid metabolism. Glycolysis has also been shown to be essential for the development and proliferation of murine DCs (Kratchmarov et al., 2018). Similarly, DC-like cells differentiated from mouse BMDMs using GM-CSF also showed higher glucose uptake and high mitochondrial membrane potential and oxygen consumption (Krawczyk et al., 2010).

As well as promoting monocyte differentiation to DCs, fatty acid metabolism is also essential for immunogenicity and the antigen-presenting capacity of DCs in human and mouse liver (Ibrahim et al., 2012). And recently, it has been reported that, apart from glucose, DCs store glycogen and express enzymes required for its metabolism. Indeed, defective glycogen metabolism in DCs impaired their activation and effector functions (Thwe et al., 2017). Taken together, although DCs are phagocytes and antigen-presenting cells like macrophages, that have different metabolic requirements and utilize various signaling pathways to regulate their metabolic remodeling during cell growth and development vs. Mtb infection. While it is clear that glycolysis and fatty acid metabolism in particular play a vital role in DC function, the relevance of these metabolic pathways specifically during Mtb infection needs further investigation.

### Neutrophils

Neutrophils or polymorphonuclear (PMN) cells are the most abundant constituent of white blood cells or granulocytes and are some of the first cells to arrive at the site of bacterial infections, including TB. However, among the various phagocytes, the specific role of neutrophils during Mtb infection is less well-characterized.

Neutrophils are efficient phagocytes that also facilitate the killing of pathogens by secreting human neutrophil peptides (HNP), which are cationic proteins of the α-defensin family that bind to anionic components of plasma membranes and disrupt their integrity (Fu, 2003). After phagocytosis of Mtb, neutrophils secrete several chemokines and cytokines that attract inflammatory monocytes from the circulation (Petrofsky and Bermudez, 1999; Seiler et al., 2003; Mantovani et al., 2011). Monocyte recruitment and maturation at the site of infection can redefine the microenvironment at both metabolic and immunologic levels (Blomgran and Ernst, 2011). Studies suggest that upon mycobacterial infection, neutrophils facilitate the activation of naïve antigen-specific CD4+ T cells, triggering an adaptive immune response (Blomgran and Ernst, 2011). However, there are also reports indicating that neutrophils can act as permissive hosts for Mtb (Abadie et al., 2005; Sutherland et al., 2009; Eum et al., 2010). Depending on host- and pathogen-associated factors, and their interactions, neutrophils can also contribute to disease progression. Specifically, recruitment of neutrophils during early stages of Mtb infection has been shown to halt infection (Lyadova, 2017). However, initial activation and presence of neutrophils in lungs infected by a hypervirulent strain of Mtb resulted in exacerbation of inflammation and disease progression (Koo et al., 2012). During mycobacterial infection, neutrophils also induce transcriptional changes in macrophages leading to the generation of pro-inflammatory cytokines (Andersson et al., 2014). Alternatively, Mtb-infected, necrotic macrophages can be engulfed by neutrophils, thereby clearing cellular debris during Mtb infection. Neutrophils affect T cell development and maturation during Mtb infection by releasing IL-12 (p40 and p70), IL-10, IP-10 (interferon γ induced protein 10), and MIP-1α (macrophage inflammatory protein) (Petrofsky and Bermudez, 1999; Seiler et al., 2003; Mantovani et al., 2011). Neutrophils also communicate with DCs to deliver signals and for antigen presentation (Hedlund et al., 2010). It has been shown that the enhanced levels of GSH inside neutrophils increase the fusion of phagosomes containing Mtb with lysosomes, which inhibits mycobacterial growth (Morris et al., 2013b).

Only a few studies have explored the metabolic changes associated with neutrophil maturation and activation. One reason for the lack in this area is the relatively short life span and rapid activation of neutrophils that limit technical manipulations for long term studies. In humans, Mtb infection-induced hypoxic conditions stabilize HIF-1α in neutrophils (Ong et al., 2018). This event leads to increased secretion of matrix metalloproteases (MMP)-8 and MMP-9, which contribute to the destruction of the matrix of type1 collagen, gelatin and elastin, which are the main structural proteins of the human lung (Ong et al., 2018). In summary, more research is needed to identify the metabolic changes occurring in neutrophils and to link them to the corresponding immune responses in the context of Mtb infection.

## Mediators of Host Metabolism During Mtb Infection

**Figure 3:**
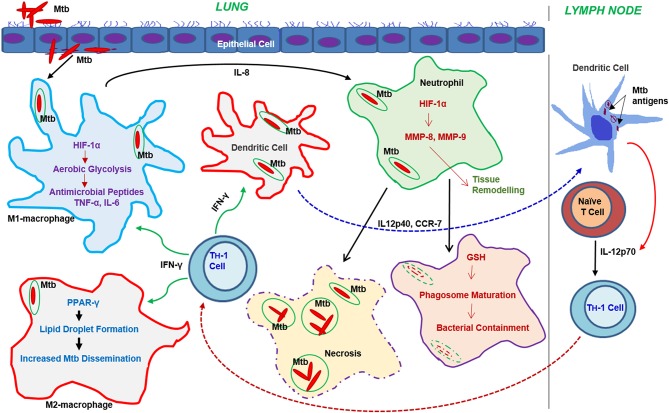
Progression of the immune response and metabolic changes in immune cells following Mtb infection. Following infection, Mtb reaches the lung where it encounters various immune cells. Macrophages and DCs are among the first immune cells to encounter Mtb. Activation of macrophages leads to the generation of antimicrobial peptides or dissemination of Mtb through the autophagy machinery. Neutrophil interaction with Mtb leads to either bacterial containment or dissemination through necrosis. DCs infected with Mtb migrate to the draining lymph nodes where they drive T cell differentiation toward a Th-1 phenotype. The activated Th-1 cells migrate back to the lungs, where they produce IFN-γ and TNF-α, which further activate macrophages leading to bacterial clearance. Metabolic reprogramming, mainly via the activation of aerobic glycolysis in macrophages, neutrophils, and dendritic cells, plays a significant role in effective functioning of these cells.

**Figure 4:**
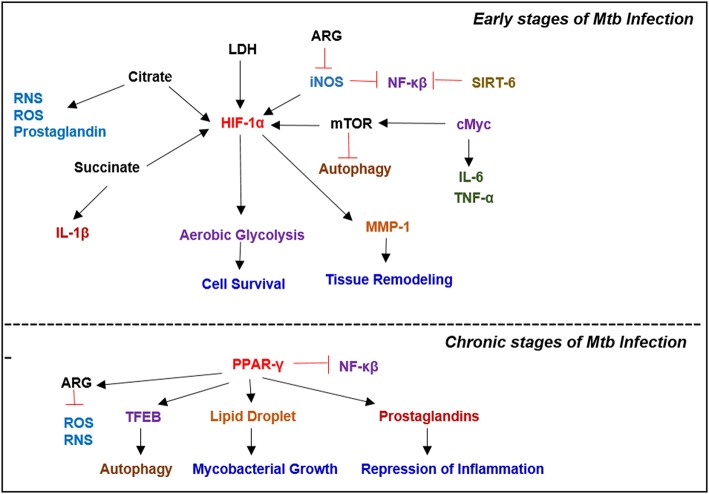
Key metabolic regulators involved in Mycobacterial infection. Infection with Mtb results in a plethora of metabolic reprogramming in immune cells leading to the induction of HIF-1α, which induces aerobic glycolysis. In the latter stages of the disease, when host immunity wanes, Mtb modulates the immune response by inducing PPAR-γ expression, which facilitates lipid droplet formation and prostaglandin synthesis, which augment bacterial survival.

Cellular signals sensed by host cells activate specific regulators that control immunometabolism (Figure 3). These signals can originate either from intrinsic stimuli of the host, such as starvation or from extrinsic factors such as microbes. Similarly, the downstream response to these stimulants shares many common molecules and pathways between homeostatic and disease conditions (Linke et al., 2017; Patsoukis et al., 2017; Krzywinska and Stockmann, 2018). The following are the key host transcriptional regulators involved in immunometabolic changes relevant for Mtb infection (Figure 4).

### HIF-1

The hypoxia-inducible factor-1 (HIF-1) is a heterodimeric transcription factor composed of two subunits: oxygen responsive HIF-1α, and constitutively expressed HIF-1β (Nizet and Johnson, 2009). In human colon and ovarian cancer cells, under non-hypoxic conditions, prolyl-1-4 hydroxylase proteins (PHDs) hydroxylate HIF-1α, which then binds von-Hippel-Lindau proteins, causing proteasomal degradation of HIF-1α (Huang et al., 1998; Semenza, 2000). Under hypoxic conditions, PHD activity is down-regulated resulting in HIF-1α accumulation and heterodimerization with HIF-1β (Semenza, 2001). This complex then binds to the promoter regions of several hypoxia-inducible genes and upregulates their expression (Corcoran and O'Neill, 2016). Hypoxia-inducible genes encode proteins involved in a myriad of cellular pathways that regulate cell survival, apoptosis, angiogenesis, erythropoiesis, glucose metabolism, and pH regulation (Semenza, 2000).

Various stimuli activate HIF-1α and, together with NF-κB, these signaling pathways regulate the immune response in hypoxic conditions (Rius et al., 2008). Under hypoxic conditions, HIF-1α fulfills the energy needs of cells by promoting aerobic glycolysis via inducing the expression of the glycolytic enzymes, such as hexokinase and phosphofructokinase (Riddle et al., 2000; Obach et al., 2004). Hypoxia and inflammation are inherently linked, as upon activation, immune cells undergo considerable metabolic reprogramming to sustain energy needs, thereby mostly switching to aerobic glycolysis. Thus, HIF-1α mediated pathways are essential in regulating immunity and inflammation. The role of HIF-1α in inducing aerobic glycolysis was elucidated in macrophages activated by LPS, and in Th-17 cells, which produce IL-17 (Tannahill et al., 2013; Gerriets et al., 2015). HIF-1α was also found to be upregulated in murine lungs upon Mtb infection inducing the Warburg effect (Shi et al., 2015).

HIF-1α is expressed in primary innate immune cells like macrophages, DCs, neutrophils, and Th-17 cells (Corcoran and O'Neill, 2016). In DCs, HIF-1α was shown to play a role in activation-induced by LPS, as blocking glycolysis using 2-DG inhibited DC maturation, measured through the reduced expression of costimulatory molecules CD80 and CD86 (Jantsch et al., 2008). A similar role for HIF-1α in macrophage activation by LPS was also demonstrated: inhibition of glycolysis using 2-DG caused a HIF-1α-mediated downregulation of IL-1β that blocked macrophage activation (Zhang et al., 2006; Tannahill et al., 2013). Additional roles for HIF-1α in macrophage differentiation and function have also been demonstrated. Specifically, HIF-1α-mediated metabolic reprogramming plays a significant role in polarization of macrophages toward the M1 or M2 phenotype (Corcoran and O'Neill, 2016). TCA cycle intermediates succinate and citrate accumulate in macrophages upon LPS stimulation, which stabilizes HIF-1α (Newsholme et al., 1986; Tannahill et al., 2013; Jha et al., 2015). Succinate increases the transcription of target gene IL-1β, whereas citrate accumulation leads to increased production of pro-inflammatory mediators- NO, ROS, and prostaglandins (Infantino et al., 2011; Tannahill et al., 2013). In neutrophils, HIF signaling leads to production of the PHD enzymes, especially PHD3, which prolong the survival of neutrophils under hypoxic conditions in both human and mice (Walmsley et al., 2011). Thus, HIF-1α plays a critical role in the homeostasis of immune cells through metabolic reprogramming during inadequate oxygen levels and also governs immune responses. In a recent study, it was shown that HIF-1α-dependent MMP-1 (collagenase) expression and secretion were synergistically upregulated by hypoxia during Mtb infection in MDMs and normal human bronchial epithelial cells (NHBFs) (Belton et al., 2016). They also showed that HIF-1α is stabilized by Mtb even in the absence of hypoxia (Belton et al., 2016). Consistent with this, in mice models of Mtb infection, IFNγ-activated macrophages exhibited increased HIF-1α levels (Braverman et al., 2016). In a zebrafish model of *M. avium* infection, increasing HIF-1α levels were associated with increased bacterial killing (Elks et al., 2013). Similarly, HIF-1α deficiency in the myeloid lineage blocked IFNγ-induced killing of Mtb (Braverman et al., 2016).

HIF-1α, which regulates half of the transcriptional responses to IFNγ, induces the cellular metabolic shift from OXPHOS to aerobic glycolysis in IFNγ-activated macrophages by inducing LDH (lactate dehydrogenase) activity to catalyze lactate production, and by limiting acetyl CoA for the TCA cycle (Nagao et al., 2019). HIF-1α also regulates the inflammatory response and NO production in IFNγ-activated macrophages against infection. Indeed, lack of HIF-1α reduces ATP levels and NO production at the inflammatory sites (Nagao et al., 2019). In contrast, HIF-2α reduces the levels of RNS in infected neutrophils and opposes control of bacterial burden (Elks et al., 2013). The IFNγ signaling cascade involves the induction of iNOS, which reduces the expression of RelA (NF-kB family), thereby negatively regulating the inflammatory response (Braverman and Stanley, 2017). This activity of iNOS has been suggested to stabilize the HIF-1α-induced immune response in IFNγ-activated macrophages during Mtb infection (Braverman and Stanley, 2017).

### mTOR

The mammalian target of rapamycin (mTOR) belongs to the PI3K- related serine/threonine protein kinase family and comprises two distinct molecular complexes: mTOR complex1 (mTORC1) and mTOR complex 2 (mTORC2) (Laplante and Sabatini, 2012; Singh and Cuervo, 2012). mTOR is involved in balancing anabolic vs. catabolic reactions to maintain cellular homeostasis. mTOR is a master regulator of a myriad of cellular pathways and functions, including protein synthesis, metabolism, disease progression, and cell death (Saxton and Sabatini, 2017). mTOR phosphorylates the kinase p70S6 kinase1 (S6K1) (Holz et al., 2005). Upon activation, S6K1 phosphorylates and activates several downstream substrates including eIF4B (a positive regulator of the eIF4F complex) and PDCD4 (an inhibitor of eIF4B), which promotes translation (Dorrello et al., 2006). 4EBP is an essential cellular regulator of translation. It inhibits translation by binding and sequestering eIF4E, thereby preventing assembly of the eIF4E complex. Upon phosphorylation by mTORC1 at multiple sites, 4EBP dissociates from eIF4E, and 5′ cap-dependent mRNA translation is facilitated (Brunn et al., 1997; Gingras et al., 1999). mTOR is also involved in *de novo* lipid synthesis through the transcription factor, sterol responsive element binding protein (SERBP), which controls the expression of genes involved in fatty acid and cholesterol biosynthesis (Singh and Subbian, 2018). mTORC1 also promotes nucleotide biosynthesis by inducing ATF-4 dependent methylenetetrahydrofolate dehydrogenase (NADP+ dependent) 2 (MTHFD2) expression, which is a vital component of the mitochondrial tetrahydrofolate cyclase that provides one-carbon units for purine biosynthesis (Ben-Sahra et al., 2016). mTORC1 also activates CAD (carbamoyl- phosphate synthetase), a key component required for pyrimidine synthesis (Ben-Sahra et al., 2013; Robitaille et al., 2013). mTOR also increases HIF-1α translation and thus is involved in the metabolic shift to aerobic glycolysis (Saxton and Sabatini, 2017). In addition, the Mtb-induced metabolic switch of the host cellular machinery toward aerobic glycolysis is mediated by a TLR-2-dependent pathway in human monocytes and macrophages, which requires the AKT-mTOR axis (Lachmandas et al., 2016a).

Together with positively regulating anabolic processes, mTOR also suppresses catabolic processes to promote cellular growth. One of the major cellular processes regulated by mTOR is autophagy. mTOR phosphorylates, and thereby inactivates ULK1, which is a kinase that triggers autophagy by forming a complex with ATG13, FIP2000, and ATG10, to drive autophagosome formation (Kim et al., 2011). One of the survival strategies adopted by Mtb in host cells is to subvert the autophagy pathway for its benefit (Zhai et al., 2019). In mice, prolonged exposure to rapamycin before Mtb aerosol infection inhibited T cell activation and production of pro-inflammatory cytokines (Lachmandas et al., 2016a). This was also shown in human PBMCs. Studies using inhibitors have demonstrated a role specifically for mTORC1 in Th1 and Th17 activation, and associated metabolic changes; however, no role for mTORC2 has been shown in immune cell metabolic shift (Delgoffe et al., 2011). One of the signaling pathways through which mTORC1 induces metabolic reprogramming involves HIF-1α (Yecies and Manning, 2011). Using immortalized human retinal pigment epithelial cells, mTORC1 activation was shown to upregulate glycolytic genes like Glut-1, which promotes cholesterol and fatty acid synthesis involving a pathway regulated by SREBPs and PPAR-γ (Porstmann et al., 2008). In contrast, mTORC2 activates metabolic reprogramming through Myc and AKT in the human breast cancer cell line MCF-7 (Sarbassov et al., 2005; Zou et al., 2015).

### cMyc

The cellular myelocytomatosis (cMyc) is a member of a proto-oncogene family of transcription factors involved in regulating cell proliferation (Stine et al., 2015). Structurally, cMyc consists of an N-terminal transactivation domain and a C-terminal basic helix-loop-helix (HLH) leucine zipper (LZ) domain, which binds to a CACGTG E-box DNA sequence (Meyer and Penn, 2008). This binding facilitates the recruitment of histone acetyltransferase and elongation factors that can modify the transcriptional response of several genes. Deregulated and sustained Myc expression is a hallmark of cancer cells, and it constitutively activates a cell growth program in an mTOR-dependent manner (Dang, 2011). Myc controls metabolic reprogramming by binding to open chromatin of target genes involved in glycolysis and glutaminolysis, which promotes their efficient transcription. Myc dimerizes with Max, which is also a DNA-binding helix-loop-helix leucine zipper protein, to alter gene expression (Stine et al., 2015).

Upon mycobacterial infection of human PBMCs with different pathogenicity *M. bovis* (BCG), *M. avium, M. kansasii*, and *M. chelonae*, the Wnt/beta-catenin signaling pathway was found to stimulate cMyc through the MAPK/ERK pathway, resulting in the upregulation of key cytokines, including TNF-α and IL-6, which inhibit mycobacterial growth (Yim et al., 2011). In this context, Myc played a role in the anti-mycobacterial response without influencing cell proliferation or changing the G0/G1 cell cycle phase of the macrophages.

### Sirtuins (SIRTs)

Sirtuins are a family of seven proteins with deacetylase activity that requires NAD^+^ for their function (Sauve and Youn, 2012). Generally, sirtuins modulate cellular processes by inhibiting NFκB signaling and associated pro-inflammatory responses in several clinical conditions associated with chronic and acute inflammation and other metabolic disorders such as diabetes and obesity (Kauppinen et al., 2013; Vachharajani et al., 2016). TLR4 stimulation in THP-1 macrophages leads to a sirtuin-mediated metabolic shift from enhanced glycolysis to increased fatty acid oxidation, which typically delays early inflammation (Liu et al., 2012). In addition, the role of sirtuins in modulating the host immune response toward a more anti-inflammatory response during Mtb infection has been reported (Cheng et al., 2017). Mtb infection down-regulates SIRT1 expression in THP-1 macrophages, as well as in mice lung, which downregulates the expression of the RelA/p65 units of NFκB and promotes inflammatory resolution (Cheng et al., 2017). Furthermore, in contrast to an earlier observation, it has been shown that inhibition of SIRT6 increases the expression of glucose transporters such as GLUT1 and GLUT4, which promote glucose absorption and glycolysis in a mouse model of type 2 diabetes (Sociali et al., 2017). Another study reported increased SIRT6 expression during the early stages of Mtb infection in murine macrophages, which suppresses both pro-inflammatory and other anti-microbial responses at this early stage of infection (Shi et al., 2019). This observation suggests that clinical intervention or host-directed therapeutic strategies that regulate availability of sirtuins might help control disease by promoting a pro-inflammatory immune response.

### PPARs

PPAR-γ is a member of the ligand-activated transcription factor family (Theocharis et al., 2004). It forms a heterodimer with the retinoid X receptor (RXR) that binds to specific PPAR-response elements (PPREs) on the promoter regions of target genes (Kota et al., 2005). Expression of PPAR-γ can be activated by lipid metabolites; PPAR-γ also modulates lipid and glucose metabolism, and other cellular functions such as proliferation, differentiation, and inflammation (Almeida et al., 2012). Mtb infection increases PPAR-γ gene expression in host immune cells, which downregulates NF-κB signaling and enhances prostaglandin (PG) E2 production that represses pro-inflammatory cytokines and Th1 responses (Almeida et al., 2012; Mahajan et al., 2012). Increased PPAR-γ expression in Mtb-infected macrophages has also been associated with lipid droplet formation (Mahajan et al., 2012; Salamon et al., 2014). The accumulated lipids in these infected cells provide nutrients and promote bacterial growth. In addition, this PPAR-γ mediated lipid accumulation positively correlates with increased expression of scavenger receptors, including macrophage receptor with collagenous structure (MARCO), macrophage scavenger receptor (MRS), and CD36 in macrophages within human leprosy lesions (Cruz et al., 2008). These findings suggest that PPAR-γ promotes intracellular lipid accumulation by modulating the expression of several genes involved in lipid absorption as well as fatty acid synthesis during Mtb infection.

PPAR-α is another isoform of the PPAR family. It is a transcription factor that binds to *cis*-acting DNA elements and regulates the expression of several genes involved in lipid and glucose metabolism (Rakhshandehroo et al., 2010). PPAR-α regulates mitochondrial as well as peroxisome function and thus energy metabolism (Kersten, 2014). It enhances fatty acid oxidation and ketogenesis, whereas it inhibits fatty acid synthesis and glycolysis (Mandard et al., 2004). Thus, PPAR-α activation helps prevent lipid accumulation in Mtb-infected cells. PPAR-α activation also upregulates expression of transcription factor EB (TFEB) and promotes autophagy as well as host innate immunity during Mtb infection (Kim et al., 2017). The induction of TFEB also promotes lipid catabolism, which reduces intracellular Mtb growth in bone marrow-derived macrophages (Kim et al., 2017).

### Arginases

Arginases are involved in the final steps of the urea cycle, where they convert arginine to ornithine and urea. Arginases exist in two isoforms; a cytosolic Arginase 1(ARG1) and a mitochondrial Arginase 2 (ARG2). Arginases compete with nitric oxide synthase (NOS) for a common substrate, arginine (Mori, 2007). While inducible NOS promotes a pro-inflammatory response, ARG is associated with the anti-inflammatory response in host immune cells (Yang and Ming, 2014). Thus, a shift in arginine metabolism is associated with the outcome of both innate and adaptive immune responses. ARG1 is predominantly present in M2 macrophages, which are mainly localized in the periphery of granulomas (Mattila et al., 2013). Increased expression of ARG1 in macrophages suppresses their antimicrobial activities by downregulating the production of nitric oxide (NO) and reactive RNS. In contrast, another study suggested a host-protective role for ARG1 in hypoxic TB granulomas: ARG1 regulated T cell proliferation and hyper-inflammation that controlled tissue necrosis and inflammatory lung pathology in murine TB model granulomas (Duque-Correa et al., 2014). This study underscored the crucial role of ARG1 in controlling inflammation and necrosis in hypoxic granulomas, where NO is ineffective. Similar to ARG1, ARG2 also has been shown to downregulate NO production and restrict an effective immune response during *Helicobacter pylori* infection (Lewis et al., 2011). This process involved the positive regulation of ARG2 by the liver X receptor, shifting arginine metabolism toward polyamine synthesis to exert an anti-inflammatory response in macrophages. In the same study, the authors also reported a decreased bacterial burden in *Agr2*^−/−^ mice as compared to wild type, which was associated with increased NO production in the absence of ARG (Lewis et al., 2011). Thus, the metabolic shift mediated by ARG1 and 2 exerts an anti-inflammatory response in immune cells, which helps immune evasion by the pathogen.

### Inducible Nitric Oxide Synthase (iNOS)

Inducible nitric oxide synthase (iNOS) catalyzes the production of NO, especially in macrophages activated by IFN-γ (Riquelme et al., 2013). NO is a key anti-mycobacterial molecule, and it can be converted into highly RNS such as NO3 and NO2 within Mtb-infected macrophages (Jamaati et al., 2017). In addition to its microbicidal activity, NO also acts as a secondary messenger and modulates the expression of genes involved in IFN-γ signaling pathways during Mtb infection (Lee and Kornfeld, 2010; Herbst et al., 2011). HIF-1α is an important factor for IFN-γ-mediated control of Mtb infection (Braverman et al., 2016). NO positively regulates HIF-1α expression and contributes to the protective macrophage response against Mtb infection (Braverman and Stanley, 2017). Furthermore, NO inhibits prolonged NFκB activation in macrophages, and limits excessive inflammation and tissue damage during Mtb infection (Braverman and Stanley, 2017). Thus, iNOS and its product NO exert direct antimicrobial responses and also modulate IFN-γ-mediated anti-Mtb activities and the inflammatory response during infection. In addition, increased NO leads to nitrosylation of iron-sulfur proteins present in the electron transport chain, inhibiting mitochondrial respiration (Brown, 2001). Nitrosylation of cysteine residues of enzymes involved in glycolysis, TCA cycle, and fatty acid oxidation, alters their activity and modulates both glucose and fatty acid metabolism in activated immune cells (Doulias et al., 2013; Kelly and O'Neill, 2015). It has been shown that NO production in LPS-activated macrophages and DCs is associated with mitochondrial dysfunction and a shift toward increased glycolysis (Everts et al., 2012). Overall, NO produced during infection causes nitrosylation of enzymes involved in glucose and fatty acid metabolism, which modulates the host immune response to infection.

### Sphingolipids

The mucus secreted by lung alveolar epithelial cells acts as the first line of defense against microbial infections (Sharma and Prakash, 2017). One of the major components of these surfactants are sphingolipids, which play a crucial role in protecting the host against invasion of macrophages by Mtb (Garg et al., 2004). Sphingolipids are regulated by the *de novo* sphingolipid biosynthesis pathway and the sphingomyelin catabolic pathway (Gault et al., 2010). Specific sphingolipids associated with inflammatory and metabolic diseases are sphingomyelin (SM), ceramide (Cer), ceramide-1 phosphate (C1P), sphingosine (Sph), sphingosine-1 phosphate (S1P), and lactosylceramide (LacCer). All metabolites of sphingolipid biosynthesis are metabolically associated with each other to maintain a state of equilibrium within the innate immune cells and cope with the lipid overload produced during inflammatory disorders (Sharma and Prakash, 2017). The reversible/interconvertible nature of these metabolites establishes their roles as physiological (cell membrane integration) and functional (macrophage-activation) components of cells to maintain homeostasis (Ohanian and Ohanian, 2001). S1P, one of the most studied sphingolipids produced by the enzymatic activity of sphingosine-1 kinase (SK1), signals through five G-coupled receptors (S1PRs). Among these S1PRs, S1PR-1, 2, and 3 are immune regulators involved in pathogen control (Sanchez and Hla, 2004). Induction of S1PR2 has been shown to enhance the anti-mycobacterial activity of alveolar macrophages. SK1 activity is also important for phagosome maturation, innate activation of macrophages, and control of Mtb replication. Mtb is capable of inhibiting SK1 activity leading to a decrease in Ca^2+^ ion concentration that can block phagolysosome maturation (Malik et al., 2003). This process is facilitated by the increased levels of sialylated glycosphingolipid content in epithelial lung cells due to environmental or genetic factors. In pulmonary TB patients, the low serum level of S1P is suggested to be the result of reduced activity of SK1 by Mtb. Enhanced S1P levels are also shown to promote phagosome maturation and reduce the lung Mtb burden, thus improving histopathology in mouse models of infection (Anes et al., 2003; Garg et al., 2004). While S1P and C1P are known to promote cell repair, division, and survival, other sphingolipids such as ceramide are involved in cell death and inflammation (Sawai and Hannun, 1999). Ceramides are also suggested to induce drug resistance against rifampin in Mtb clinical isolates, and establish persistent survival of Mtb (Speer et al., 2015). This prolonged survival of Mtb is also linked with the S1P-pAKT-mediated upregulation of mTOR signaling in macrophages (Liu et al., 2009, 2010). During Mtb infection, FM formation indicates lipid overload in the host cells (Russell, 2011). To maintain sphingolipid balance in FM, saponin C (SapC) degrades glycosphingolipids to ceramide; SapC also mediates transfer of Mtb lipid antigen from intra-lysosomal membranes to CD1b to activate antigen-specific T cells (Kim et al., 2010). Upregulation of SapC seen in granulomas justifies the accumulation of LacCer, an important intermediate of sphingolipid metabolism found in abundance in the caseum compartment of granulomas, but only in trace amounts in other regions/cells (Kolter and Sandhoff, 2005, 2006). Since FM favors Mtb growth, accumulation of sphingolipid and its metabolites indicates altered phagolysosome biogenesis in Mtb-infected host cells. This lipid overload in granulomas also suggests a shift in lipid metabolism conferred by infection, which is crucial for Mtb survival.

## Epigenetic Regulation of Immunometabolism

Epigenetic modifications can be induced by nutritional states of cells, which drive the inflammatory response accompanying metabolic disease (Barres et al., 2013). Mechanisms by which epigenetic modulations occur, include (I) Genomic DNA methylation by DNA methyltransferases including DNMT1, DNMT3A, and DNMT3B, which generally results in transcriptional repression, (II) histone modifications including acetylation, methylation, phosphorylation, ubiquitylation and sumoylation (Bannister and Kouzarides, 2011; Raghuraman et al., 2016). Immune regulation through methylation changes is reported in TNF-α, UBASH3B (Ubiquitin-associated and SH3 domain-containing protein B) and TRIM3 (Tripartite motif-containing-3) genes (Wang et al., 2010; Hermsdorff et al., 2013). Role of altered DNA methylation in regulating immune function of T cells and macrophages is well documented in metabolic disorders, including obesity and T2DM (Barres et al., 2013; Raghuraman et al., 2016). Macrophage differentiation from monocytes is associated with various histone-modifying components involving H3K4me1, H3K4me3, and H3K27ac at promoter and enhancer regions (Saeed et al., 2014). Besides, cytokine production by macrophages is induced by H3K4me3 and demethylation of H3K27 (Takeuch and Akira, 2011). Also, HDAC3 regulates inflammatory genes in macrophages, while HDAC2 resolves inflammation by suppressing IL-6 (Chen et al., 2014; Zhang et al., 2015). The role of H3 acetylation in regulating inflammatory genes in LPS stimulated monocytes has also been reported (Iglesias et al., 2012).

Increased glycolysis and β-oxidation lead to accumulation of acetyl CoA which is used as a group donor by histone acetyltransferases (HAT) leading to relaxation of chromatin structure resulting positive regulation of transcription (Laker and Ryall, 2016). Similarly, NAD^+^ was found to control epigenetic remodeling in monocytes through SIRT1 during acute systemic inflammation (Davis and Gallagher, 2019). The chromatin remodeling in BCG stimulated innate immune cells is primarily due to histone modification (H3K4me3 and H3K9me3) in the glycolysis genes leading to activation of AKT/mTOR/HIF1α pathway, which induces a metabolic shift in host cells from OXPHOS to aerobic glucose metabolism. This finding shows the rewiring of cellular metabolism to facilitate increased production of cytokines such as TNF-α and IL6 that promote mycobacterial killing (Kleinnijenhuis et al., 2011; Arts et al., 2016).

Mtb tends to prevent the expression of immune regulatory genes such as CIITA, CD64, and HLA DR through altering chromatin dynamics and modifying histones at specific promoter sites (Pattenden et al., 2002). In blood cells from patients with pulmonary tuberculosis, DNA hypermethylation of CpG sites in TLR2 promoter was observed (Chen et al., 2014). In another study, using global histone acetylation (Ac)/methylation (Me) in blood leukocytes of pulmonary tuberculosis patients showed H3K14 hypoacetylation and H3K27 hypermethylation played a role in developing active pulmonary tuberculosis (Chen et al., 2017). Mtb (Rv1988) can interfere with the host genetic remodeling also by inducing histone modifications (H3) of non-tail arginine of H3 to alter the antigen presentation of macrophages (Yaseen et al., 2015). In a recent study, it was observed that Mtb infection in DCs led to induction of stable DNA demethylation at enhancers elements across the genome (Pacis et al., 2019).

## Concluding Remarks

Recent advances in TB research, including investigations into the role of metabolic reprogramming in different immune cells, have expanded our understanding of the pathogenesis and progression of the disease. It is now widely recognized that the immune response and metabolic remodeling are interconnected. Therefore, the net host response to Mtb infection is a result of combined immunologic and metabolic activities of the immune cells. However, much more work is needed to elucidate the intricate interactions among various metabolic programming pathways, which can tip the balance in the survival advantage toward the host or Mtb. Similarly, the causal link between immune response and metabolic remodeling needs further exploration, including investigations on how Mtb interferes with these host processes. A comprehensive understanding of the immunometabolic processes will not only help in our understanding of how pathogenic bacteria subvert immune response in their favor, but will also aid in uncovering new treatment/vaccination strategies to control TB more effectively. In this regard, the components of glycolysis and oxidative phosphorylation reactions associated with Mtb infection of immune cells could be harnessed for designing host-directed treatment strategies to enhance bacterial clearance and improve treatment outcomes.

## Author Contributions

LS, SS, YB, and ST conceived the concept. RK, PS, AK, and SS wrote the manuscript. PS and AK contributed equally. All authors read, edited, and agreed to publish the manuscript.

### Conflict of Interest

The authors declare that the research was conducted in the absence of any commercial or financial relationships that could be construed as a potential conflict of interest.
